# Supplementary figures and images for: Identification and pathogen detection of a Neocypholaelaps species (Acari: Mesostigmata: Ameroseiidae) from beehives in the Republic of Korea
Source: PLoS One. 2024 Apr 11;19(4):e0300025. doi: 10.1371/journal.pone.0300025 (PMC11008822; doi:10.1371/journal.pone.0300025)

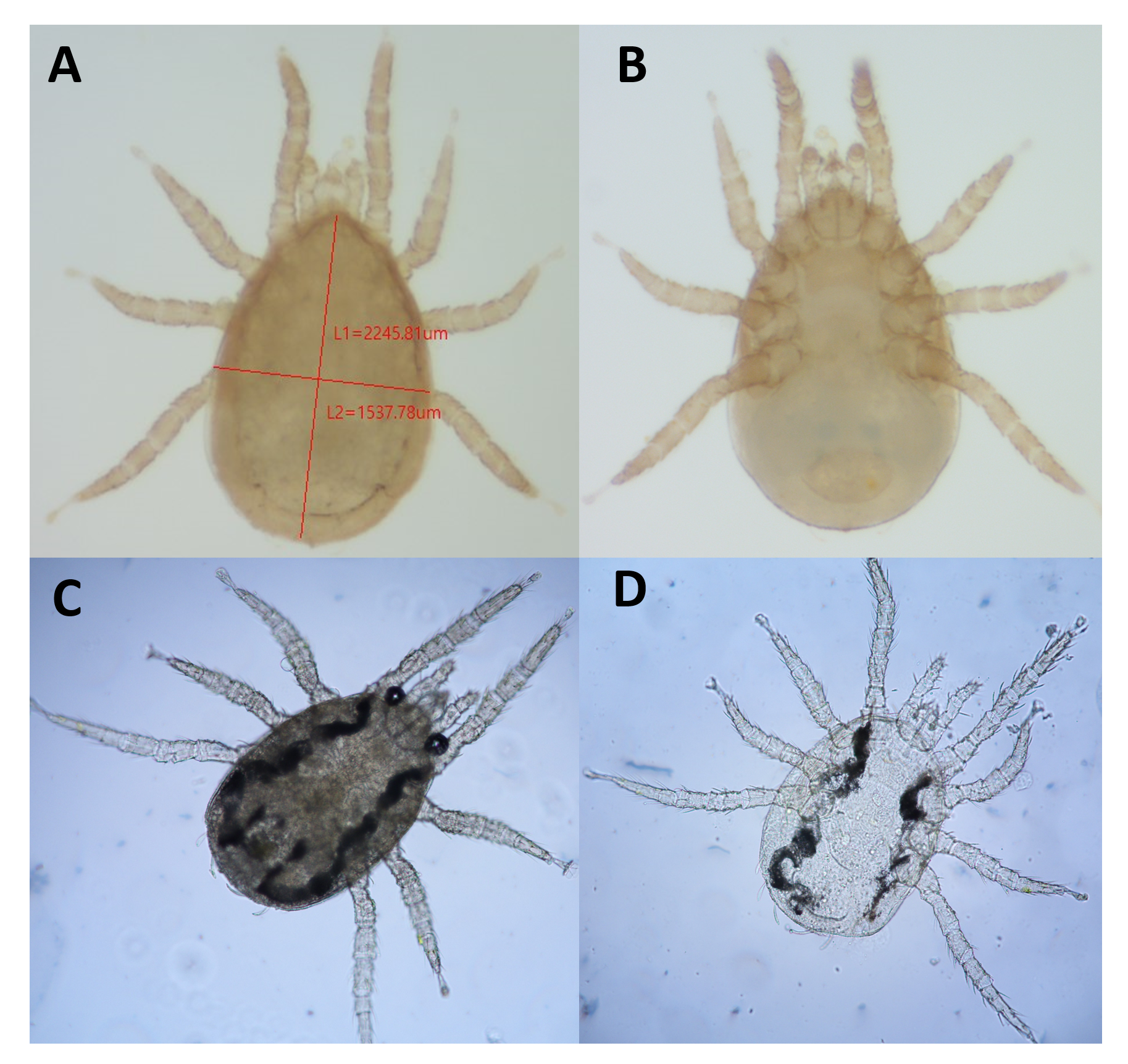

Supplement: S1 Fig — (A, B) Optical microscope Discovery V8 Stereo (Germany) with a magnification of 5.0× (Dorsal and ventral of Neocypholaelaps sp. adult mite). (C, D) Dorsal and ventral view of Neocypholaelaps sp. adult mite under a Leica DM1750M microscope at 10× magnification. (TIF) [file pone.0300025.s001.tif]

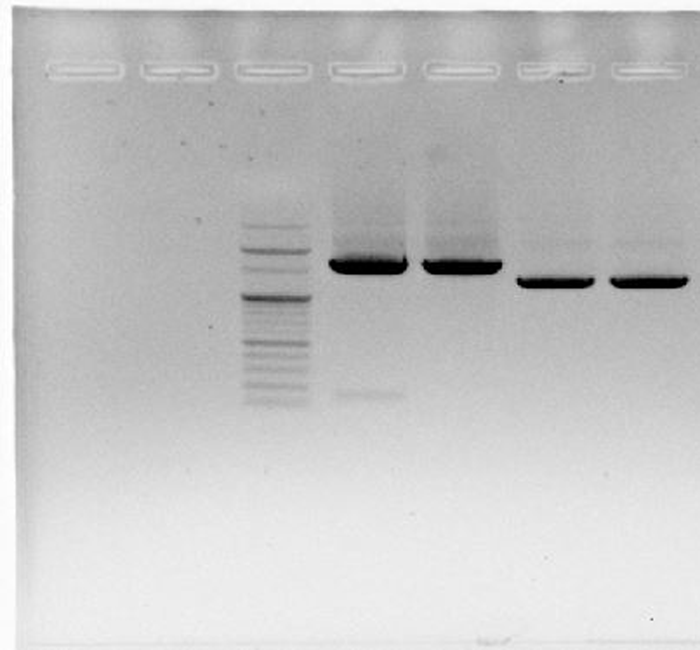

Supplement: S1 Raw image — (TIF) [file pone.0300025.s003.tif]
