# Supplementary material for: Identification and pathogen detection of a Neocypholaelaps species (Acari: Mesostigmata: Ameroseiidae) from beehives in the Republic of Korea
Source: PLoS One. 2024 Apr 11;19(4):e0300025. doi: 10.1371/journal.pone.0300025 (PMC11008822; doi:10.1371/journal.pone.0300025)
Supplement: S1 Table — (DOCX) [file pone.0300025.s002.docx]

**S1 Table. Primers and probes used for detection of honeybee pathogens**

| No. | Target | Primer | Sequence (5ꞌ→3ꞌ) | Amplicon size (bp) | Reference |
| --- | --- | --- | --- | --- | --- |
| 1 | AFB | AFB-F | AAATCATCATGCCCCTTATG | 158 | [1] |
|  |  | AFB-R | CGATTACTAGCAATTCCGACT |  |  |
|  |  | Probe | FAM-CGTACTACAATGGCCGGTACAACG–BHQ-1 |  |  |
| 2 | EFB | EFB-F | TGTTGTTAGAGAAGAATAGGGGAA | 69 |  |
|  |  | EFB-R | CGTGGCTTTCTGGTTAGA |  |  |
|  |  | Probe | Cy5-AGAGTAACTGTTTTCCTCGTGACGGT-BHQ-3 |  |  |
| 3 | ASP | ASP-F | GCTGCCCATCAAGCACGG | 127 |  |
|  |  | ASP-R | CCTACAGAGCGGGTGACAAAG |  |  |
|  |  | Probe | JOE-TGTGTGTTGGGTCGTCGTCCCCTCTC–BHQ-1 |  |  |
| 4 | ASCO | ASCO-F | ATTGCGCCCTCTGGTATTC | 215 |  |
|  |  | ASCO-R | CCACTAGAAGTAAATGATGGTTAGA |  |  |
|  |  | Probe | TexasRed-GCTTGAGGGTTGCAATGACGCTCG BHQ-2 |  |  |
| 5 | *Spiroplasma* sp. | Ms-160 F | TTGCAAAAGCTGTTTTAGATGC | 189 | [2] |
|  |  | Ms-160 R | TGACCAGAAATGTTTGCTGAA |  |  |
| 6 | *Spiroplasma* *apis* | S.apis ITS F | AATGCCAGAAGCACGTATCC | 190 |  |
|  |  | S.apis ITS R | GAACGAGATATACTCATAAGCTGTTACAC |  |  |
| 7 | *Nosema apis* | N.apis F | CAATATTTTATTGTTCTGCGAGG | 241 | [3] |
|  |  | N.apis R | AAAGTCTATTGTATTGCGCGTGCT |  |  |
| 8 | *Nosema* *ceranae* | N.ceranae F | CAATATTTTATTATTTTGAGAGA | 223 |  |
|  |  | N.ceranae R | TATATCTATTGTATTGCGCGTGCA |  |  |
| 9 | *Trypanosoma* spp. | Try-RT-F | GGGCGGCATATCTGTTACAC | 276 | [4] |
|  |  | Try-RT-R | AAGAGCCGACATCGAAGGAT |  |  |
| 10 | AmFV | AmF-For | CACGAAAGCCCTCAAATCGT | 155 |  |
|  |  | AmF-Rev | GCGGGTAAATCCACTAAGGC |  |  |
| 11 | SBV | SBV-F | AGAAGACATTTGATACAGTGGACTC | 131 | [1] |
|  |  | SBV-R | GGAATTCCAGATTCTTCGTCCAC |  |  |
|  |  | Probe | FAM–GATTTGTTTAATGGTTGGGTTTCTGGTA–BHQ-1 |  |  |
| 12 | BQCV | BQCV-F | CCTTTGGCAATAGAACAAATACC | 143 |  |
|  |  | BQCV-R | GTGGCTATATCGAGATTATTCCG |  |  |
|  |  | Probe | Cy5- AGTCGCAGAGTTCCAAATACCGTACTATG- BHQ-3 |  |  |
| 13 | CBPV | CBPV-F | CGCAAGTACGCCTTGATAAAGAAC | 101 |  |
|  |  | CBPV-R | ACTACTAGAAACTCGTCGCTTCG |  |  |
|  |  | Probe | Cy5- TCAAGAACGAGACCACCGCCAGTTC- BHQ-3 |  |  |
| 14 | ABPV | ABPV-F | TGCCCTATTTAGGGTGAGGAG | 239 | [1] |
|  |  | ABPV-R | GGAGTTTCCACATCATGAAAGG |  |  |
|  |  | Probe | FAM- CTCTGAAGAAAACTCAGTTGAAACGGAAC- BHQ-1 |  |  |
| 15 | IAPV | IAPV-F | TGCCCTATTTAGGGTGAGGAG | 245 |  |
|  |  | IAPV-R | GGAGTTTCCACATCATGAAAGG |  |  |
|  |  | Probe | ROX- ACTAGTGAGAACTCGGTTGAGACCCAAG- BHQ-2 |  |  |
| 16 | KV | KV F | ATGTTGCAGCTTCGGCTTTC | 466 | [5] |
|  |  | KV R | GGGTCCAATTCGTTCCT |  |  |
| 17 | VDV1- DWV | VDV-1DWV-F | CATGGAAATGGGATCAAAC | 206 | [6] |
|  |  | VDV-1DWV-R | AAACGGGCTGAAAATCACAC |  |  |
| 18 | VDV-1 | VDV-1 F | GCCCTGTTCAAGAACATG | 413 | [7] |
|  |  | VDV-1 R | CTTTTCTAATTCAACTTCACC |  |  |
| 19 | DWV | DWV-F | TTCAACTCGGCTTTCTACGG | 170 | [1] |
|  |  | DWV-R | GTGTCTTTTTCTCTTTCTGACACC |  |  |
|  |  | Probe | ROX- ATGTCAACATTGGTATGCTCCGTTGAC- BHQ-2 |  |  |
| 20 | KSBV | KSBV F | GACCAAGAAGGGAATCAG | 123 |  |
|  |  | KSBV R | CATCTTCTTTAGCACCAGTATCCA |  |  |
|  |  | Probe | HEX- CCACATAGATTCCTGCCCGCGA- BHQ-1 |  |  |
| 21 | KBV | KBV-F | ACCAGGAAGTATTCCCATGGTAAG | 79 |  |
|  |  | KBV-R | TGGAGCTATGGTTCCGTTCAG |  |  |
|  |  | Probe | HEX- CCGCAGATAACTTAGGACCAGATCAATCACA- BHQ-1 |  |  |
| 22 | Lake Sinai virus | LSV1-For | GGCCGTCATGGTGGCGAATAAAATCGTCGCTGTTGTCCTTG | 198 | [8] |
|  |  | LSV1-R | CAACCTCTCGTAAGCAAGCC |  |  |
|  |  | LSV2-RT-F | CCATGTTGTTGATCCGGCTCTGGGAGCGTC | 188 | [4] |
|  |  | LSV2-RT-R | ACGGGCTGAGTTGGCGGTACTTCACGCATA |  |  |
|  |  | LSV3-RT-F | CCACATCATTGAGCCGGGTGTGGGAGCGAT | 188 |  |
|  |  | LSV3-RT-R | ACGGACTTAGCTGCAGGTACCTGTTGCATA |  |  |
|  |  | LSV4-RT-F | CCACGTCGTTGAGCCTGATTTGGGAATCGC | 188 |  |
|  |  | LSV4-RT-R | GAGGGCTGAGTTGTAGGTACTTGTTGCATA |  |  |

Abbreviations: AFB: American foulbrood; EFB: European foulbrood; ASCO: Ascosphaera apis; ASP: Aspergillus flavus; SBV: sacbrood virus; KSBV: Korea sacbrood virus; DWV: deformed wing virus; BQCV: black queen cell virus; KBV: Kashmir bee virus; ABPV: acute bee paralysis virus; IAPV: Israeli acute paralysis virus; AmFV: Apis mellifera filamentous virus; LSV1: Lake Sinai virus 1; LSV2: Lake Sinai virus 2; LSV3: Lake Sinai virus 3; LSV4: Lake Sinai virus 4; VDV-1: Varroa destructor virus 1; VDV1-DWV: Recombinant Varroa destructor virus 1 and deformed wing viruses.

**Reference**

1. Truong A-T, Yoo M-S, Seo SK, Hwang TJ, Yoon S-S, Cho YS. Prevalence of honey bee pathogens and parasites in South Korea: A five-year surveillance study from 2017 to 2021. Heliyon. 2023;9(2). <https://doi.org/10.1016/j.heliyon.2023.e13494>
2. Meeus I, Vercruysse V, Smagghe. Molecular detection of Spiroplasma apis and Spiroplasma melliferum in bees. J Invertebr Pathol. 2012;109(1): 172–4
3. Vanengelsdorp D, Evans JD, Saegerman C, Mullin C, Haubruge E, Nguyen BK, et al. Colony collapse discorder: a descriptive study. PLoS One. 2009;4(8):e6481. <https://doi.org/10.1371/journal.pone.0006481>
4. Nguyen T-T, Yoo M-S, Truong A-T, Lee JH, Youn SY, Lee S-J, et al. First identification of *Tyrophagus curvipenis* (Acari: Acaridae) and pathogen detection in *Apis mellifera* colonies in the Republic of Korea. Sci Rep. 2023;13(1):9469. <https://doi.org/10.1038/s41598-023-36695-z> PMID: 37301922
5. Fujiyuki T, Takeuchi H, Ono M, Ohka S, Sasaki T, Nomoto A, et al. Kakugo virus from brains of aggressive worker honeybees. Adv Virus Res. 2005;65:1–27.
6. Zioni, N., Soroker, V. & Chejanovsky, N. Replication of *Varroa destructor* virus 1 (VDV-1) and a *Varroa destructor* virus 1–deformed wing virus recombinant (VDV-1–DWV) in the head of the honey bee. Virology. 2011; 417: 106–12.
7. Radzevičiūtė, R, Theodorou P, Husemann M, Japosshvili G, Kirkitadze G, Zhusupbaeva A, et al. Replication of honey bee-associated RNA viruses across multiple bee species in apple orchards of Georgia, Germany and Kyrgyzstan. J Invertebr Pathol. 2017; 146: 14-23.
8. Runckel, C, Flenniken ML, Engel JC, Ruby JG, Ganem D, Andino R, et al. Temporal analysis of the honey bee microbiome reveals four novel viruses and seasonal prevalence of known viruses, Nosema, and Crithidia. PLoS One. 2011; 6: e20656, <https://doi.org/10.1371/journal.pone.0020656>
