# Supplementary material for: Identification and pathogen detection of a Neocypholaelaps species (Acari: Mesostigmata: Ameroseiidae) from beehives in the Republic of Korea
Source: PLoS One. 2024 Apr 11;19(4):e0300025. doi: 10.1371/journal.pone.0300025 (PMC11008822; doi:10.1371/journal.pone.0300025)
Supplement: Attachment — Submitted filename: Response to Reviewers.docx [file pone.0300025.s004.docx]

**Responses to Reviewers’ Comments**

*We appreciate the reviewers for their invaluable comments. As explained below, we have revised our original manuscript and materials in response to all of the reviewers’ comments. What follows are our point-by-point responses to the comments.*

**Reviewer #1**: In this study, Neocypholaelaps of the genus were found in Western honeybee hives in Korea (ROK). Based on molecular analysis, the authors believe the mite belongs to a new species. Furthermore, the detection of bee pathogens in this mite sample highlights its importance in the spread of bee diseases. Regarding the idea that this species is new, I think the authors should clarify it further and resubmitting it in another article. The following is my opinion:

Title:

Identification and pathogen detection of a Neocypholaelaps species (Acari: Mesostigmata: Ameroseiidae) from beehives in the Republic of Korea

Response: We sincerely appreciate your insightful feedback on the potential novel species identified in our study. The title was revised according to the suggestion.

“Identification and pathogen detection of a *Neocypholaelaps* species (Acari: Mesostigmata: Ameroseiidae) from beehives in the Republic of Korea”

Specific comments:

Abstract:

Line 22: Add the scientific name of the host

Response: The scientific name of the host (*Apis mellifera*) has been included in the updated manuscript.

Line 26-27: It is recommended to delete the description about the new species

Response: Modified as suggested.

Introduction:

Line 39-41: “Although some mite species…..” -> A very ambiguous explanation with no further references to support it.

Response: The sentence has been clarified as follow: In *A. mellifera,* some mite species may not exhibit parasitic behavior (e.g. Tracheal, *Forcellinia faini, Melichares dentriticus, Afrocypholaelaps aficana* mites*)* [1–4]. Additionally, specific mite species have been identified as potential vectors of disease to bees (e.g. *Varroa* and *Tropilaelaps* mites) [5–7], while a notable number of mite species can induce stress in bees (e.g. *Neocypholaelaps indic* and *Caroglyphus lactis* mites) [8,9]. This clarification is supported by relevant references (Lines 38–42)

Line 48: small honeybees -> do you mean dwarf honey bee? Add the scientific name of the bee

Response: The sentences has been revised as suggested: Dwarf honeybees (*Apis florea)*

Line 49: Add the scientific names of the giant honey bees

Response: Sentences were added as suggested: Giant honeybees (*Apis dorsata)*

M &M

Line 87-88: Add the scientific name of the mite host

Response: Modified as suggested. The scientific name of the mite host was honeybee (*Apis mellifera*)

Line 88-89: The description of collection method is very vague. The preservation method of pathogen samples is usually not the same as that of identification samples stored in 95% ethanol. This will almost cause the pathogen samples to degrade in a short period.

Response: In the methods section at Lines 90 to 94 and Lines 132 to 134, we improved the description of the collection method and elucidated the procedures as follows:

*Neocypholaelaps* sp. KOR23 mites were observed on beehive of *A. mellifera* colonies in Wonju City, Gangwon Province, ROK, in 2023. Nine colonies from three apiaries were used to collect *Neocypholaelaps* sp. KOR23 mites. These mites were gathered from hive debris and stored in 50 mL Falcon containing 95% ethanol that was labeled with hive number. The sample was immediately subjected to total nucleic acid (TNA) extraction upon arrival the laboratory. (Lines 90–94)

The TNA extracted from the mite samples was tested for honeybee pathogens [25,49]. The honeybee pathogens that were detected in Korean honeybee colonies were employed to assess their presence in *Neocypholaelaps* mite. These pathogens include the following viral pathogens. (Lines 132–134)

Line 101-102: Although the sampling method refers to the previous paper, the explanation here is too brief and difficult to understand.

Response: the description for nucleic acid extraction was added in the manuscript

“The hive debris samples were observed and pooled to collect *Neocypholaelaps* mite under microscope Discovery V8 Stereo. Three samples were collected from three apiaries. Ten adult mites from each apiary were transferred onto petri dish containing UltraPure™ distilled water (Invitrogen, USA) for washing. Mites were manually collected for extraction of TNA using a mounting needle under a dissecting microscope. Those mites were pooled and placed in a 2 mL Eppendorf tubes containing 300 µL phosphate-buffered saline (1x) and 2.381 mm steel beads (Hanam, ROK) and used for TNA extraction [25]. The TNA of *Neocypholaelaps* mite used keep in –20°C for PCR amplification of *CO1,* 18S, and 28S regions and checking honeybee pathogens.” (Lines 102–110).

Line 114: The types and primers of pathogen detection should refer to previous papers, but only the types of pathogen detection are mentioned here.

Response: Sentences were added more information as suggested: Honeybee pathogens were detected using RT-qPCR Kits [iNtRON Biotechnology, Inc., ROK], Pobgen bee pathogen detection Kit [Postbio, ROK], and iTaq Universal SYBR Green One-Step Kit [Bio-Rad, USA]). The sequences of primers targeting honeybee pathogens for detection in *Neocypholaelaps* mite samples are provided in Supplementary S1 Table. (Lines 141–145).

The S1 Table was added in supporting information at Line 459 **“S1 Table. Primers and probes used for detection of honeybee pathogens”**

Line 122-123: It is unclear why the author did not detect VDV-1 and LS1 viruses, but to detect VDV-DWV.

Response: The *Varroa destructor virus-*1 (VDV-1) and Lake Sinai Virus 1 (LSV1) pathogens were tested and added more information in Table 2.

Line 133: Relevant sequences should be deposited into the GenBank and given accession numbers.

Response: Thank you for your suggestion. The 18S and 28S genes of *Neocypholaelap*s sp. KOR23 were deposited in an NCBI database with accession number: OR576776.1 and OR576780.1 (Lines 149–151)

Line 136-148: It is a bit far-fetched to define a new species in morphology. In the morphological identification, there is no problem with genus-level identification, but there is no description or comparisons at the species level. Even the details in the figures can only be barely identified to the genus. For species-level, the morphological identification did not provide corresponding hand drawings or detailed microscopic photos.

Response: The sentence mentioning the genus level of morphological identification was added in Lines 161 to 168. For species identification the molecular method relying on CO1, 18S, and 28S DNA was used.

“The morphological identification of the collected mite showed similar characteristics to the genus of *Neocypholaelaps* adult mite as describe of *N. favus* Ishkawa, 1968, *N. apicola* Delfinada-Baker & Baker, 1983, *N. geonomae* n. sp. Moraes & Narita, 2010, and *N. indica* Evans, 1963 [27–29,37]. *Neocypholaelaps* adult mite dorsal and ventral surfaces were observed under both microscope discovery V8 Stereo (S1A and S1B Fig) and electron microscopy Leica DM1750M (S1C and S1D Fig) for magnification. The genus *Neocypholaelaps* is characterized by its extremely small size, approximately 2060–2190 μm in length and 1322–1537 μm in width (S1A and S1B Fig).”

L160 said that based on the morphological characteristics, they designed 18S and 28S primers. The explanation is unclear and I can’t understand the correlation?

Response: revision was done for the sentence to clarify the intended meaning in the material and methods section from Lines 113 to 121

“Based on the morphological characteristics results observed under the microscope, the *Neocypholaelaps* mite was identified in this study. Then, the genetic analysis of *CO1,* 18S, and 28S genes was done for further identification of collected mites. Primers were designed based on the sequence information of *Neocypholaelaps* species available on GenBank and BOLD systems (Table 1). The available primers of *CO1* gene were designed for *N. indica* Evans (1963), *N. apicola* Delfinado-Baker & Baker, 1983, and *Neocypholaelaps* sp. (NCBI accession nos.: LC522089.1, KP966315.1, and MF911280); The 18S and 28S gene were designed for *Neocypholaelaps* sp. (NCBI accession nos.: FJ911807.1 and FJ911742.1).”

Line 175-183: The DNA extraction method and analysis method described in the M&M are rough. It seems that 10 mites are used to form a DNA sample of KOR23. For the genetic analysis, it seems that only two sequences from other Neocypholaelaps species are used. These trees can explain the correct identification of the genus, but the data explain the new species is scarce. Furthermore, the species description of APGD-2010 is also unclear, and it is difficult to explain whether there are any difference in other aspects, such as in morphology.

Response: Thank you for your comments, we revised the last sentence of the paragraph to be more appropriate with the result identified in this study: “Our study newly provides the genetic information of a strain in the genus *Neocypholaelaps* isolated from beehives in the ROK in 2023 and was designated as *Neocypholaelaps* sp. KOR23”. In addition, the term “new species” is removed from the whole manuscript.

Line 194 and 198: Two sentences conflict with the results of fungal testing

Response:

Thank you for your suggestion. Those sentences were corrected at Lines 221 to 229. “The identities of the pathogens were confirmed by real-time RT-PCR (Table 2). Nine honeybee pathogens (AFB, ASCO, *Spiroplasma* sp., DWV, BQCV, CBPV, IAPV, KV, and LSV3) were detected in this mite species out of a total of 25 that were screened. Results indicated the presence of bacteria and viruses in *Neocypholaelaps* sp. KOR23 with high *Ct* values. Some honeybee pathogens findings suggest that *Neocypholaelaps* sp. KOR23 has the potential to harbor pathogens and may serve as a vector for honeybee infections, contributing to the rapid increase in disease outbreaks among honeybee colonies in the ROK. However, *Nosema apis,* *N. ceranae,* and *Trypanosoma* spp. have not been identified in *Neocypholaelaps* sp. KOR23”.

Discussion

There is a little description and discussion of the pathogen detections. Whether there is relevant pathogen data for the mite species may be used for discuss and comparison.

Response: Sentences were added for discussion about the pathogen detection in this section from Lines 267 to 278 “Possibly, this could be one of the factors contributing to severe damage to honeybee colonies. This study is the first to demonstrate the presence of honeybee pathogens in *Neocypholaelaps* sp. mite. Recent research has also reported the appearance of honeybee pathogens in parasites from honeybee colonies. Almost honeybee pathogens have been detected in *Varroa* mite [5,6,54,55]; DWV and BQCV were found on the small hive beetle (*Aethina tumida*)[56]; DWV and *Trypanosoma* spp. were detected on *Tyrophagus curvipenis* [25]. Some viral and bacterial pathogens of honeybee were detected in *Neocypholaelaps* sp. KOR23. However, microsporidian (*Nosema apis* and *N. cernaena)* and protozoa (*Trypanosoma* spp.) were not seen in *Neocypholaelaps* sp. KOR23. Further study on the detection of the same pathogens in the honeybee colonies where the mites were detected might be needed to confirm the role of *Neocypholaelaps* sp. KOR23 mite in pathogen transmission.”

Figures:

Fig 1: L137 writes the body length and width of Fig1B. The scale should be increased or the length should be marked in this figure. The rest of Fig1 (A,C,D) is unclear, especially A, which feels very redundant.

Figure descriptions should be reconsidered, for example, Figures 1 and 2 do not show explanations of "morphological characteristics”

Fig2 should be added in the caption as female (or maybe another gender or age?)

Response: Thank you for your comment and suggestion about the morphology figure. We moved the Fig 1 to Supplementary data as S1 Fig, and Fig 2 was changed to be Fig 1 in the revised manuscript. The adult female was added in the caption as commented

**Fig 1. Morphological characteristics of *Neocypholaelaps* sp. adult female mite.** Identity of acaroid mites was determined by analyzing morphological characteristics using a phase contrast microscope. (A) Dorsal view of the mite. (B) Ventral view of the mite.
