# Supplementary material for: Identification and pathogen detection of a Neocypholaelaps species (Acari: Mesostigmata: Ameroseiidae) from beehives in the Republic of Korea
Source: PLoS One. 2024 Apr 11;19(4):e0300025. doi: 10.1371/journal.pone.0300025 (PMC11008822; doi:10.1371/journal.pone.0300025)
Supplement: Attachment — Submitted filename: Response to Reviewers.docx [file pone.0300025.s005.docx]

**Responses to Reviewers’ Comments**

*We appreciate the reviewers for their invaluable comments. As explained below, we have revised our original manuscript and materials in response to all of the reviewers’ comments. What follows are our point-by-point responses to the comments.*

**Reviewer #1:** There are minor issues should be check. Please refer and consider making appropriate revisions. I think it can be accepted in the future.

L40: delete the word “Tracheal,”

Response: I deleted it.

L41, L43: possibility of spelling errors on scientific names “Afrocypholaelaps aficana”, “Neocypholaelaps indic” and “Caroglyphus lactis”, please check.

Response: These have been corrected as follows:

“Afrocypholaelaps aficana” 🡪 “Afrocypholaelaps africana”

“Neocypholaelaps indic” 🡪 “Neocypholaelaps indica”

“Caroglyphus lactis” 🡪 “Carpoglyphus lactis”

L45-53: In this paragraph, four genera of mites are described as being particularly harmful to bees, but a very abrupt description of Tyrophagus is added in L53-54.

Response: I thank the reviewer for the reasonable comment. I corrected these sentences more logically and consistently in lines 46-56.

“Obligate honeybee parasites like *Varroa* (Varroidae, Mesostigmata), *Euvarroa* (Varroidae, Mesostigmata), *Tropilaelaps* (Laelapidae, Mesostigmata), and *Acarapis* (Tarsonemidae, Prostigmata inflict direct harm and facilitate disease spread [10–12]. These mites have co-evolved with their hosts, developing an intimate dependence on honeybees for survival. While *Varroa* mites primarily parasitize bees in their brood cells [6,13], *Euvarroa* and *Tropilaelaps* demonstrate host specificity towards Dwarf honeybees (*Apis florea)* and giant honeybees (*Apis dorsata)*, respectively [14–18]. The most pathogenic species within the genus *Acarapis* primarily parasitize *A. mellifera* but have also been found in Asian honeybee species [19–23]. Interestingly, the genus *Tyrophagus* (Acaridae: Sarcoptiformes) adds to the list of potentially detrimental mites, displaying parasitic behavior detrimental to both honeybees and bumblebees [24,25].”

L55-56: states that Neocypholaelaps are mainly distributed in tropical regions, but most of the species later exemplified in paragraphs L56-69 are distributed in subtropical and temperate regions. Further possible reason or additional explanations should be added.

Response: We have included this information in the introduction (lines 57-68) and the discussion section (lines 253-259)

L75&77: The terminology of L75&77 "higher level classification" may be worth discussing. The author may have intended to express a classification closer to the genus or species level, but the term may also be easily confused with the advanced classification methods used to describe family or above levels.

Response: This has been corrected (Line 79).

“However, these genes offer limited information for classifying *Neocypholaelaps* species within the genus”

L93 “50 mL Falcon”-> “50 mL tubes (Falcon)”

Response: I corrected it.

L244 "with several reported subspecies" It would be better if there are relevant literature guidance

Response: I have included this information in lines 259-263.

L261-263: It can be described that KOR23 is densely populated and may cause harm to bee colonies, but rather than saying "leading to damage", perhaps it is more conservative to say "leading to threaten" or "leading to weaken"? It can also be slightly blended with the conclusion of the last paragraph of the discussion.

Response: This expression has been corrected with further research needed to confirm the damage of *Neocypholaelaps* mite infestation (Lines 279-281).
